# Platelet-Rich Plasma Versus Polydeoxyribonucleotide for Myofascial Pain Syndrome and Musculoskeletal Disorders: A Narrative Review of Mechanisms, Clinical Evidence, and Translational Considerations

**DOI:** 10.3390/bioengineering13091045

**Published:** 2026-09-08

**Authors:** Se Yeong Jeon, Chul Hee Jung, Seok Yeon Choi, Dong Ha Lee

**Affiliations:** 1 Science and Technology Department, Ulsan National Institute of Science and Technology, Ulsan 44919, Republic of Korea; 2 Department of Orthopaedic Surgery, Aerospace Medical Center, Republic of Korea Air Force, Cheongju 28187, Republic of Korea; 3 Department of Rehabilitation Medicine, Aerospace Medical Center, Republic of Korea Air Force, Cheongju 28187, Republic of Korea; 4 Department of Orthopaedic Surgery, Seoul National University Hospital, Seoul 03080, Republic of Korea

**Keywords:** platelet-rich plasma, polydeoxyribonucleotide, myofascial pain syndrome, trigger point, trapezius, rhomboid, regenerative medicine, adenosine A2A receptor, tendinopathy, injection therapy

## Abstract

Myofascial pain syndrome (MPS) of the cervicoscapular girdle—most typically involving the upper trapezius and the rhomboid muscles—is among the most frequent causes of chronic regional musculoskeletal pain, yet the mainstays of interventional treatment (dry needling, local anesthetic trigger point injection, corticosteroid, botulinum toxin) act principally on nociceptive transmission or on the contractile taut band rather than on the disordered tissue biology that sustains it. Two injectable biologics with fundamentally different design logic have been proposed to fill this gap. Platelet-rich plasma (PRP) is an autologous, polypharmacological concentrate that delivers a supraphysiological bolus of platelet α-granule growth factors and leukocyte-dependent immunomodulators. Polydeoxyribonucleotide (PDRN) is a standardized, allogeneic-source (salmonid sperm DNA) low-molecular-weight deoxyribonucleotide mixture that acts through a single defined molecular target, the adenosine A2A receptor, supplemented by nucleoside salvage. This narrative review contrasts the two agents across four axes: (i) molecular and cellular mechanism; (ii) the biological plausibility of each in the specific microenvironment of the myofascial trigger point (MTrP)—an acidic, hypoxic, sensitizer-rich focus; (iii) the current clinical evidence in MPS and, by extension, across tendinopathy, plantar fasciitis, knee osteoarthritis, and acute muscle injury; and (iv) practical translational considerations including standardization, regulatory status, cost, and trial design. We conclude that PRP has the broader and methodologically stronger clinical evidence base in tendon and joint indications but is undermined by preparation heterogeneity and by consistently negative results in acute muscle injury, whereas PDRN offers reproducibility, an anti-inflammatory and pro-angiogenic profile that maps plausibly, based on preclinical evidence, onto MTrP pathophysiology, though direct validation in human myofascial tissue is lacking, and an excellent safety record, but is supported almost entirely by small, short-horizon, and frequently non-randomized studies. Neither agent can currently be recommended as standard care for trapezius or rhomboid MPS. We propose a mechanistic rationale and a concrete trial framework, including muscle-specific ultrasound-guided delivery, pressure pain threshold and Neck Disability Index as co-primary endpoints, and mandatory injectate characterization, for the head-to-head studies that the field now requires.

## 1. Introduction

Myofascial pain syndrome (MPS) is a regional pain disorder defined by the presence of myofascial trigger points (MTrPs): hyperirritable nodules within a palpable taut band of skeletal muscle that reproduce the patient’s familiar pain on compression and characteristically refer pain to a distant zone [1]. It is one of the most common causes of non-articular musculoskeletal pain encountered in primary care, rehabilitation medicine, orthopaedics, and pain clinics [2,3]. Its cost is measured not in structural failure but in persistence: patients frequently cycle through analgesics, physical therapy, and repeated needling procedures over months to years, with incomplete or short-lived relief [2].

The cervicoscapular girdle is the epicentre of clinical MPS. The upper trapezius is the single most frequently implicated muscle, and the rhomboid major and minor, lying deep to it and functionally coupled to scapular control, are a common and often overlooked generator of interscapular pain [4,5]. This anatomical layering has direct therapeutic consequences: a needle placed in the superficial trapezius does not treat a rhomboid MTrP, and in a prospective randomized double-blind comparison, ultrasound-guided injection targeting the rhomboid major produced greater improvement in pain, disability, and quality of life than superficial trapezius injection in patients with MPS [5]. Sonographic guidance has therefore become central to modern trigger point intervention, both for accuracy and for the avoidance of pneumothorax in the interscapular region [6].

Against this anatomical precision, the pharmacology of trigger point injection has remained comparatively crude. The classical comparator trial by Kamanli and colleagues, comparing lidocaine injection, botulinum toxin injection, and dry needling in MPS, established the template that still dominates practice: all three arms improved, none was decisively superior, and the mechanism of benefit was attributed largely to mechanical disruption of the taut band and to interruption of nociceptive input rather than to biological repair [7]. None of these agents is designed to correct the ischemic, acidic, inflammatory microenvironment that contemporary pathophysiological models place at the centre of MTrP persistence.

Two injectable biologics have been advanced to address precisely that gap, and they embody opposite design philosophies. Platelet-rich plasma (PRP) is an autologous blood derivative in which platelets—and, depending on preparation, leukocytes—are concentrated above baseline; on activation, α-granules release a broad and partially uncontrolled cocktail of growth factors including platelet-derived growth factor (PDGF), transforming growth factor-β (TGF-β), vascular endothelial growth factor (VEGF), and insulin-like growth factor, together with chemokines and interleukins [8]. Polydeoxyribonucleotide (PDRN) is, by contrast, a defined and standardized pharmaceutical: a mixture of deoxyribonucleotide polymers of 50–1500 kDa purified from the sperm DNA of *Oncorhynchus mykiss* (salmon trout) or *Oncorhynchus keta* (chum salmon) by a process that removes active proteins and peptides, and whose principal mechanism is engagement of a single receptor, the adenosine A2A receptor, supplemented by provision of nucleosides to the purine salvage pathway [9].

PRP is thus a polypharmacological, patient-variable, autologous product; PDRN is a monopharmacological, batch-standardized, xenogeneic-source drug. Clinicians in Korea, Italy, and increasingly elsewhere now choose between them routinely for the same patient with refractory trapezius or rhomboid pain, largely on the basis of cost, reimbursement, and personal experience rather than on comparative evidence.

The purpose of this narrative review is fourfold: [Terminological note: “PDRN” refers specifically to low-molecular-weight deoxyribonucleotide polymer preparations (50–1500 kDa; A2A agonist mechanism), whereas “polynucleotide (PN)” denotes higher-molecular-weight preparations used for viscosupplementation, which may differ in mechanism and clinical profile. This distinction is maintained consistently throughout.] to set out the mechanistic contrast between PRP and PDRN at a level of detail sufficient to generate testable hypotheses; to assess how well each mechanism maps onto the specific biology of the MTrP; to synthesize the clinical evidence for both agents in MPS and in the wider musculoskeletal indications from which MPS practice is largely extrapolated; and to identify the design features that future head-to-head trials will need in order to be informative.

## 2. Methods

This is a narrative, not a systematic, review, and it is reported in that spirit. PubMed/MEDLINE, Scopus, and Google Scholar were searched from database inception to April 2026 using combinations of the terms “platelet-rich plasma”, “PRP”, “polydeoxyribonucleotide”, “PDRN”, “polynucleotide”, “myofascial pain syndrome”, “trigger point”, “trapezius”, “rhomboid”, “tendinopathy”, “plantar fasciitis”, “osteoarthritis”, and “muscle injury”. Reference lists of retrieved reviews and trials were hand-searched. Preference was given to randomized controlled trials (RCTs), systematic reviews and meta-analyses, and mechanistic studies published in indexed, peer-reviewed journals; case reports were cited only where they represent the entirety of the available evidence for an indication. Non-English publications and conference abstracts without full text were excluded. Because the evidence base for PDRN in particular is dominated by small and non-randomized designs, no formal risk-of-bias scoring or quantitative synthesis was attempted; instead, study design and sample size are reported explicitly throughout so that the reader may weight the findings accordingly. The combined searches identified approximately 340 potentially relevant citations; after title and abstract screening, 89 full-text articles were assessed, of which approximately 52 were included (approximately 37 primary studies, 11 systematic reviews or meta-analyses, and 4 mechanistic studies). Inclusion criteria: English-language peer-reviewed publications on PRP or PDRN in myofascial pain, tendinopathy, plantar fasciitis, osteoarthritis, or muscle injury; and mechanistic studies on A2A receptor agonism or platelet growth-factor biology. Exclusion criteria: conference abstracts without full text; non-English publications; studies confined to aesthetic or non-musculoskeletal indications.

## 3. Biological Rationale

### 3.1. The Trigger Point Microenvironment: What an Injectate Would Have to Fix

The dominant model of MTrP formation—the integrated hypothesis—proposes that excessive acetylcholine release at the motor endplate produces sustained sarcomere shortening and a localized contracture, which compresses local capillaries, generates ischemia and hypoxia, and precipitates a local energy crisis [4,5,6,7,8,9,10,11,12,13]. This is not merely a mechanical account; the compressed, hypoxic focus becomes biochemically distinct.

The decisive evidence came from in vivo microdialysis. Using a 32-gauge microanalytical needle, Shah and colleagues sampled the interstitial milieu of active MTrPs in the upper trapezius and demonstrated significantly elevated concentrations of protons (i.e., lower pH) together with substance P, calcitonin gene-related peptide (CGRP), bradykinin, serotonin, norepinephrine, tumor necrosis factor-α (TNF-α), and interleukin-1β compared with latent MTrPs, normal muscle, and remote uninvolved sites [10,11]. Subsequent work confirmed that these abnormalities extend beyond the palpable nodule and are accompanied by measurable changes in tissue architecture and perfusion: Doppler and elastographic ultrasound show MTrPs as focal hypoechoic regions with increased stiffness and altered local blood flow [12]. Electrophysiological studies localize spontaneous electrical activity to the extrafusal motor endplate and attribute local tenderness principally to peripheral nociceptor sensitization, with referred pain reflecting central convergence [13]. These electrophysiological and biochemical findings underscore that MTrP pathophysiology extends beyond local tissue biology: peripheral nociceptor sensitization drives central sensitization, and the resulting expansion of receptive fields explains referred pain and allodynia. Any pharmacological intervention targeting MTrP-associated pain must therefore be evaluated not only for effects on local tissue repair and inflammation, but also for capacity to modulate peripheral nociceptive signalling—an axis on which PDRN (via A2A-mediated attenuation of neurogenic inflammation) and PRP (via potential modulation of nerve growth factor) have theoretical, though unproven, relevance.

Three features of this microenvironment are relevant to injectate selection.

First, it is hypoxic and hypoperfused. Any agent that promotes angiogenesis and restores local perfusion addresses an upstream driver rather than a downstream symptom.

Second, it is inflammatory in a neurogenic sense. The elevation of TNF-α, IL-1β, substance P, and CGRP indicates that suppression of pro-inflammatory cytokine signalling is mechanistically attractive, and, conversely, that an injectate which itself provokes cytokine release may be counterproductive in a tissue that is already sensitized.

Third, it is fascially connected. The MTrP does not exist in isolation from its investing fascia and from adjacent myofascial planes [14], which is the rationale behind the interfascial plane blocks that have shown efficacy in cervicoscapular and dorsal MPS, including rhomboid intercostal and erector spinae plane approaches [15,16,17]. Injectate volume and fascial distribution, not only pharmacology, may therefore contribute to effect.

Practically, these considerations mean that trigger point delivery in the trapezius and rhomboid should be ultrasound-guided, both to reach the intended muscle layer [5,18] and to permit accurate reporting of what was actually injected where, a requirement that has been met inconsistently in the literature reviewed below.

### 3.2. Platelet-Rich Plasma: Polypharmacology by Design

PRP is produced by centrifugation of autologous anticoagulated whole blood to concentrate platelets in a reduced plasma volume. Its therapeutic premise is that platelet α-granules, on activation, release a coordinated repertoire of growth factors—PDGF, TGF-β1, VEGF, epidermal growth factor, fibroblast growth factor, and insulin-like growth factor-1—that recruit and activate progenitor cells, stimulate angiogenesis, and drive extracellular matrix synthesis [8].

The central and unresolved problem of PRP is heterogeneity. Multiple classification systems exist, none validated, and they variously stratify products by platelet concentration, leukocyte content, fibrin architecture, and activation method [8]. The most clinically consequential axis is leukocyte content. Leukocyte-rich PRP (LR-PRP) delivers neutrophils and monocytes along with platelets, amplifying early inflammatory signalling and matrix metalloproteinase activity; leukocyte-poor or “pure” PRP (LP-PRP) minimizes this. Current synthesis suggests LR-PRP may be preferable in tendinopathy, where a controlled inflammatory stimulus appears to assist a failed healing response, whereas LP-PRP is favoured in intra-articular cartilage applications, where leukocyte-driven catabolism is undesirable [8,19]. A systematic review and meta-analysis of knee osteoarthritis found LP-PRP to be associated with a more favourable adverse-reaction profile than LR-PRP, although clinical outcome differences were less clear-cut [20].

For MPS specifically, this distinction is not academic. If the MTrP milieu is already characterized by elevated TNF-α and IL-1β [10], injecting a leukocyte-rich product into it is a mechanistically ambiguous act: it is mechanistically plausible—though unproven in the clinical MPS context—that LR-PRP could add to rather than resolve the sensitising load; equally, it may provoke the resolution phase of a stalled inflammatory cascade. The near-total absence of injectate characterization in published MPS studies of PRP means that this question remains entirely open.

A second problem is inter-patient and intra-patient variability. Baseline platelet count, hematocrit, age, medication (notably antiplatelet agents), and the specific commercial kit and spin protocol all shift the final composition [8,19]. Two patients receiving “PRP” for trapezius MPS may receive substantially different biological products, and the same patient may receive different products at successive sessions. This is a fundamental obstacle to dose–response reasoning and to reproducible trial results.

### 3.3. Polydeoxyribonucleotide: Monopharmacology by Design

PDRN occupies the opposite position. It is a mixture of deoxyribonucleotide polymers of defined molecular weight range (50–1500 kDa) extracted from salmonid sperm DNA under a controlled purification and sterilization process that guarantees the absence of active proteins and peptides capable of provoking an immune reaction [9].

Its mechanism has two arms. The primary and better-characterized arm is agonism at the adenosine A2A receptor. In vitro and in vivo studies consistently show that the effects of PDRN are abolished by the A2 antagonist 3,7-dimethyl-1-propargylxanthine, which has greater affinity for A2A than for A2B, implicating A2A specifically; the current model holds that PDRN acts as a prodrug, generating active deoxyribonucleotides, nucleosides, and bases that engage the receptor [9,21]. A2A engagement is a well-established anti-inflammatory and pro-angiogenic signal. In collagen-induced arthritis, PDRN reduced pro-inflammatory cytokine production and disease severity in an A2A-dependent manner [22]. In experimental spinal cord injury, PDRN reduced TNF-α and IL-1β release, limited apoptosis, and stimulated Wnt/β-catenin-driven neurogenesis, with the Wnt effects abolished by adenosine receptor blockade [21]. In a rat pressure ulcer model, PDRN reduced oxidative stress and accelerated healing [9]. Across models, A2A activation upregulates VEGF and promotes neovascularization [9,23].

The second arm is the nucleoside salvage pathway. By supplying nucleosides and purine bases to tissue in which de novo synthesis is energetically expensive, PDRN supports nucleic acid synthesis in cells operating under metabolic stress—a property first demonstrated in cultured human fibroblasts, where PDRN increased protein synthesis and proliferation [23]. Hypoxic, energy-depleted tissue is precisely the setting in which this matters.

The mechanistic fit with MTrP biology is, based on preclinical and indirect evidence, biologically plausible, though direct validation in human myofascial tissue is absent: an agent that simultaneously suppresses TNF-α and IL-1β, upregulates VEGF and restores perfusion to a hypoperfused focus, and supplies substrate to cells in an energy crisis is addressing three of the four canonical elements of the integrated hypothesis. Whether this translates into clinical benefit is a separate question, addressed in Section 4.

PDRN’s safety profile is a genuine comparative advantage. Post-marketing surveillance covering more than 300,000 dispensed prescriptions over five years confirmed an excellent safety record, attributed in large part to the absence of effects on the immune system [9]. Unlike corticosteroids, PDRN carries no risk of tendon weakening, fat pad atrophy, skin depigmentation, or hyperglycemia [24,25].

### 3.4. Direct Mechanistic Comparison

Table 1 summarizes the mechanistic contrast between the two agents across the dimensions most relevant to MPS and musculoskeletal applications.

The table makes the trade-off explicit. PRP offers biological breadth at the cost of reproducibility; PDRN offers reproducibility and a mechanistically coherent anti-inflammatory/pro-angiogenic profile at the cost of biological breadth. Which trade is preferable is an empirical question, and it is likely to have different answers in different tissues—a tendon requiring a coordinated multi-growth-factor matrix remodelling response is not the same target as a sensitized, hypoperfused muscle nodule.

## 4. Clinical Evidence

### 4.1. Myofascial Pain Syndrome of the Trapezius, Rhomboid, and Cervicoscapular Region

This is the indication of primary interest and, unfortunately, the one with the thinnest direct evidence for either agent.

For PRP, no adequately powered randomized controlled trial has evaluated injection into the upper trapezius or rhomboid for MPS. The available signal is indirect and comes from two sources. The first is the broader trigger point injection literature, in which needling itself—independent of injectate—produces meaningful short-term benefit; a meta-analysis of seven RCTs of trigger point dry needling for plantar heel pain confirmed benefit over control, illustrating the magnitude of the non-specific needling effect against which any injectate must be judged [26]. The second is the small comparative literature on other injectates in chronic MPS, including a retrospective series of ultrasound-guided dextrose injection [27], which demonstrates that clinically useful outcomes are achievable but does not isolate the contribution of the injected substance.

For PDRN, the situation is more stark: to our knowledge, no published randomized trial has evaluated PDRN injection specifically for trapezius or rhomboid MPS. Its use in this setting is extrapolated from tendinopathy and from broader musculoskeletal pain series, in which PDRN has been applied to a wide range of disorders with consistently positive but methodologically weak reporting [28].

The strongest evidence in MPS for either agent therefore comes from the masticatory system, discussed next, and its transferability to the cervicoscapular girdle should not be assumed—the masseter is a small, superficial, high-duty-cycle muscle with a distinct loading pattern and a distinct comorbidity profile (bruxism, temporomandibular disorder) compared with the postural upper trapezius or the scapular-stabilizing rhomboid.

What can be said with confidence is that delivery matters independently of the drug. The randomized double-blind comparison by Metin Ökmen and colleagues showed that ultrasound-guided rhomboid major injection outperformed superficial trapezius injection in MPS patients on pain, disability, and quality-of-life measures [5], and the interfascial plane block literature demonstrates that spread of injectate within the rhomboid–intercostal and erector spinae planes produces durable analgesia in dorsal and cervicoscapular MPS [15,16,17]. Any future PRP-versus-PDRN trial that does not standardize target muscle, needle depth, and injectate volume will be uninterpretable.

### 4.2. Masticatory Myofascial Pain: The Best Available Direct Evidence for PRP

Three studies constitute the core of the direct PRP evidence in MPS, all in masticatory muscles.

Agarwal and colleagues randomized 30 patients with clinically confirmed masseter MTrPs 1:1 to PRP injection or dry needling, with evaluation of visual analog scale (VAS) pain, range of functional movement, analgesic requirement, patient satisfaction, and sleep at 2 weeks, 1 month, and 3 months, and VAS and satisfaction at 6 months. PRP produced better pain and satisfaction outcomes than dry needling, and the authors concluded that PRP was the more effective modality for masseter MTrPs [29].

Rani and colleagues randomized 22 patients with myofascial pain dysfunction syndrome to dry needling or PRP, assessing the Pain Disability Questionnaire, numeric rating scale, maximum mouth opening, and tenderness at baseline, post-treatment, 4 weeks, and 12 weeks. Both groups improved significantly; PRP showed superior pain reduction and jaw mobility at follow-up, leading the authors to suggest PRP as the more effective long-term option [30].

A randomized controlled trial of plasma rich in growth factors in masticatory myofascial pain compared injection of a platelet-derived growth factor preparation with lidocaine injection into masseter trigger points in 50 adults, testing the hypothesis that growth-factor delivery could both relieve pain and promote muscle regeneration [31].

A fourth comparative trial placed PRP alongside botulinum toxin and local anesthesia for masseter MTrPs, situating PRP within the full range of available injectates rather than against needling alone [32].

These are encouraging results, but their limitations must be stated plainly: sample sizes of 22–50, single-centre designs, follow-up rarely exceeding six months, no injectate characterization (leukocyte content is not reported in any of them), and comparators—dry needling and lidocaine—that are themselves active. They establish that PRP is at least not inferior to conventional needling in a small superficial masticatory muscle. They do not establish superiority in the cervicoscapular girdle, and they do not permit any inference about PDRN.

### 4.3. Tendinopathy: Where PDRN Has Its Deepest, if Still Shallow, Evidence

Tendinopathy is where the PDRN literature is most developed, and it is the source of most clinical enthusiasm for the drug in Korea. [Caveat: tendinopathy differs from MTrP-bearing muscle in cellular composition, vascular architecture, and loading environment; the evidence below must be viewed as indirect support for a biologically plausible mechanism rather than a transferable clinical effect size.]

The anchor study is that of Yoon and colleagues, a case-controlled retrospective comparative study of 106 patients with chronic non-traumatic refractory rotator cuff disease unresponsive to at least one month of conservative treatment; 55 received PDRN injection and 51 continued conservative management, with follow-up to six months. The PDRN group showed significant improvement in Shoulder Pain and Disability Index, VAS, and daily analgesic consumption relative to controls, although isometric abductor strength, active range of motion, and maximal ultrasonographic tear size did not differ [33]. Ryu and colleagues reported ultrasound-guided PDRN prolotherapy for painful rotator cuff tendinopathy [34], and Do and colleagues conducted a prospective pilot study of PDRN injection in partial-thickness supraspinatus tears using ultrasound follow-up [35].

A PRISMA-compliant meta-analysis by Gwak and colleagues pooled one RCT and three retrospective observational studies of PDRN for tendon or ligament pain and found a large improvement in pain (standardized mean difference −1.43, 95% CI −1.80 to −1.06), with a still larger effect in the rotator cuff tendinopathy subgroup (SMD −2.34, 95% CI −3.61 to −1.07) [28]. The magnitude is striking; the evidence base beneath it—predominantly uncontrolled, retrospective, and unblinded—means the point estimate should be treated as hypothesis-generating rather than as an effect size to power future trials against.

The comparative context is provided by network meta-analysis. Lin and colleagues systematically compared injection therapies in rotator cuff tendinopathy across randomized trials, situating PRP, corticosteroid, prolotherapy, and other agents against one another and highlighting how sensitive the rankings are to follow-up interval and to the choice of comparator [36]. Dextrose prolotherapy has itself been tested against control injection in painful rotator cuff tendinopathy in a randomized design [37], underscoring that the “regenerative injection” field as a whole is characterized by many small trials of many agents with few direct comparisons.

In lateral epicondylitis, PDRN has been evaluated principally in case series and in combination protocols with exercise and extracorporeal shockwave therapy, with reported pain relief and normalization of common extensor tendon hypervascularity on ultrasound. The parallel autologous blood/PRP literature is more mature: Arik and colleagues randomized patients to autologous blood versus corticosteroid injection for lateral epicondylitis [38], and the broader tendon literature has established the counterpoint that argues for both biologics—glucocorticoids provide rapid short-term relief but carries documented risks to tendon integrity and inferior medium-term outcomes [39].

Table 2 summarizes the principal clinical studies of each agent in MPS and adjacent soft-tissue indications.

### 4.4. Plantar Fasciitis: The Cleanest Comparative Signal for PDRN

Plantar fasciitis provides the methodologically strongest PDRN evidence and, unusually, includes a placebo-controlled trial.

Kim and Chung randomized 40 patients with clinically diagnosed plantar fasciitis to weekly PDRN injection or normal saline for three weeks, with VAS and Manchester–Oxford Foot Questionnaire assessment at baseline, 4 weeks, and 12 weeks, and complication monitoring at 1, 2, 4, and 12 weeks. The PDRN group achieved significant improvement in both measures at 4 weeks, sustained to 12 weeks, and the authors concluded that PDRN is an effective and safe option [40]. This remains one of the few placebo-controlled demonstrations that PDRN’s effect exceeds that of needle plus fluid volume alone.

Lee and colleagues then randomized 44 patients to PDRN versus corticosteroid injection, evaluating VAS, Manchester–Oxford Foot Questionnaire, plantar fascia thickness and echogenicity on ultrasound, and complications at baseline, 1, 2, and 6 weeks, and 6 months. Corticosteroid produced greater pain relief at 2 weeks (*p* = 0.010) and 6 weeks (*p* = 0.016), but this advantage had disappeared by 6 months (*p* = 0.523) [41]. This is a clinically instructive pattern and is likely generalizable: corticosteroid front-loads relief, PDRN accrues it, and the curves converge. Given the documented risks of repeated corticosteroid exposure to tendon and fascia [39], convergence at 6 months constitutes a meaningful argument for PDRN rather than a null result.

The PRP comparator literature in plantar fasciitis is broadly consistent in shape. Tabrizi and colleagues randomized obese patients with chronic plantar heel pain to corticosteroid versus PRP, again finding an early corticosteroid advantage that did not persist [42]. Systematic reviews of prospective comparative studies have generally favoured PRP over corticosteroid at medium-term follow-up.

### 4.5. Knee Osteoarthritis: Where PRP Has the Evidentiary Advantage

Knee osteoarthritis is the indication in which both agents have been most rigorously studied and in which their evidence bases can be compared with something approaching fairness. It also demonstrates, importantly, that the two agents are not interchangeable.

For PDRN, Kim and colleagues conducted a systematic review and meta-analysis of five RCTs comparing PDRN with hyaluronic acid. PDRN produced significantly better pain improvement than HA at 1 and 2 months (*p* = 0.04 and *p* = 0.02), with no significant difference at 4 months; functional outcomes (Knee Injury and Osteoarthritis Outcome Score, Knee Society Score) did not differ at any time point, and adverse events were comparable (relative risk 2.15, 95% CI 0.17–26.67, *p* = 0.55). The authors positioned PDRN as a favourable alternative to HA for persistent pain [43]. A related but distinct product, high-molecular-weight polynucleotide (PN), was compared with high-molecular-weight HA in a double-blind, multicentre RCT of 60 patients receiving three weekly intra-articular injections, with change in weight-bearing pain at 16 weeks as the primary endpoint [44]—a reminder that “PDRN” and “polynucleotide” denote related but non-identical products with different molecular weights and different intended mechanisms (pharmacological versus viscosupplementary), a distinction frequently blurred in clinical discussion.

For PRP, the evidence is substantially larger. Dai and colleagues meta-analyzed RCTs and concluded that intra-articular PRP conferred greater benefit than HA and saline for pain relief and function at one year [45]. Belk and colleagues restricted analysis to Level 1 studies comparing PRP with HA and reached a concordant conclusion across WOMAC, VAS, and subjective IKDC outcomes [46]. Karasavvidis and colleagues found that PRP combined with HA improved pain and function relative to HA alone [47], and Zhao and colleagues reached similar conclusions regarding the combination’s efficacy and safety [48].

This literature must, however, be read alongside its most important corrective. Gazendam and colleagues, in a systematic review and network meta-analysis of randomized trials in hip osteoarthritis, found intra-articular saline injection to be as effective as corticosteroids, PRP, and HA for pain [49]. The placebo and needle-effect contribution to all intra-articular injection therapy is large, and any comparison of two active biologics without a saline arm risks confidently establishing the equivalence of two placebos.

### 4.6. Acute Muscle Injury: A Cautionary Result for PRP

Because MPS is a muscle disorder, the PRP muscle-injury literature deserves specific attention, and it is the least favourable body of evidence for PRP in the review.

Reurink and colleagues conducted a double-blind, placebo-controlled trial in three centres, randomizing 80 competitive and recreational athletes with MRI-confirmed acute hamstring injuries to intramuscular PRP or isotonic saline, with patients, clinicians, and physiotherapists all blinded. PRP did not shorten the time to return to sport [50]. Extended follow-up to one year confirmed no benefit of intramuscular PRP over placebo in return-to-play time, re-injury rate, or subjective, clinical, and MRI measures [51].

This is a well-conducted, adequately blinded, placebo-controlled negative trial in skeletal muscle—the tissue of interest in MPS. It does not refute the masticatory MPS findings, since acute traumatic muscle disruption and chronic MTrP sensitization are different pathologies with different limiting steps. But it does impose an appropriate discipline on extrapolation: the assumption that growth-factor delivery reliably improves outcomes in injured skeletal muscle is not supported by the highest-quality evidence available, and enthusiasm for PRP in muscle should be correspondingly restrained.

Table 3 summarizes the comparative evidence position across indications.

## 5. Practical and Translational Considerations

Beyond efficacy, the choice between PRP and PDRN in routine practice is shaped by logistics, regulation, and cost, and these differ enough to influence adoption independently of evidence. Table 4 summarizes the practical comparison.

Two points deserve emphasis for investigators.

First, injectate characterization must become mandatory. The single most damaging feature of the PRP literature is that “PRP” names a category, not a product. Trials should report platelet concentration relative to baseline, leukocyte and neutrophil content, activation method, spin protocol, and final volume [8,19,20]. PDRN trials, by comparison, generally report dose in milligrams, and the field should not lose this advantage.

Second, saline control arms are indispensable. The hip osteoarthritis network meta-analysis showing equivalence of saline to corticosteroid, PRP, and HA [49], and the large non-specific effect of needling itself demonstrated in the dry needling literature [26], together mean that any PRP-versus-PDRN comparison without a placebo arm can establish only relative and not absolute efficacy. In MPS in particular—where the mechanical disruption of the taut band is itself therapeutic [7]—a two-arm active comparison is at serious risk of producing an uninformative tie.

## 6. Limitations

This review has limitations that should temper its conclusions. It is narrative rather than systematic: study selection was purposive, no protocol was registered, no formal risk-of-bias instrument was applied, and no quantitative synthesis was performed. Publication bias is likely to be substantial in both studies, and is particularly concerning for PDRN, where published musculoskeletal pain studies have almost uniformly reported positive pain-reducing effects, with negative findings conspicuously absent from the record [28]. Much of the PDRN evidence is retrospective, single-centre, unblinded, and Korean or Italian in origin, raising questions about generalizability. The PRP evidence, while larger, is undermined by product heterogeneity to a degree that limits meta-analytic pooling. Most decisively, no study to date has compared PRP with PDRN head-to-head in any musculoskeletal indication; every comparative statement in this review is therefore indirect, and indirect comparison across trials with different populations, comparators, and endpoints is a weak form of inference.

## 7. Future Directions

The field needs a small number of well-designed trials more than it needs additional case series. We propose the following framework for a definitive comparison in cervicoscapular MPS.

Design. A three-arm, double-blind, randomized controlled trial: LP-PRP versus PDRN versus normal saline, all delivered under ultrasound guidance to a sonographically confirmed MTrP in a prespecified muscle (upper trapezius or rhomboid major, stratified), with identical injectate volume across arms to control for the fascial distribution effect [5,15].

Endpoints. Co-primary endpoints are pressure pain threshold at the treated MTrP (an objective, mechanistically interpretable measure) and the Neck Disability Index (a patient-centred functional measure), with VAS/NRS, analgesic consumption, sonographic MTrP area and stiffness [12], and quality of life as secondary endpoints. Follow-up should extend to at least 6 months, given the convergence pattern seen in plantar fasciitis [41].

Mandatory reporting. Full injectate characterization for the PRP arm [8,19,20]; PDRN dose in mg/mL and total dose; needle gauge, depth, and number of passes; and whether a local twitch response was elicited.

Mechanistic substudy. In vivo microdialysis of the treated MTrP before and after intervention, quantifying pH, substance P, CGRP, TNF-α, and IL-1β using the methodology established by Shah and colleagues [10,11]. This would convert the trial from a comparison of two black boxes into a test of the specific hypothesis advanced in Section 3.4—that PDRN’s A2A-mediated cytokine suppression and VEGF-driven reperfusion address the MTrP milieu more directly than growth-factor delivery does.

Beyond this, combination and sequencing strategies deserve investigation. Preclinical work has explored combining PDRN with extracorporeal shockwave therapy for muscle and tendon regeneration, with sequence-dependent effects; whether analogous synergy exists between mechanical needling, PDRN, and PRP is unknown.

## 8. Conclusions

PRP and PDRN represent two coherent but opposite answers to the same question of how to convert a symptomatic injection into a biological one. PRP maximizes biological breadth and accepts irreproducibility; PDRN maximizes reproducibility and accepts mechanistic narrowness. Across musculoskeletal indications, PRP currently holds the stronger and more rigorously tested evidence base—most clearly in knee osteoarthritis—but is contradicted by high-quality negative evidence in acute skeletal muscle injury. PDRN holds a smaller and methodologically weaker evidence base concentrated in tendinopathy and plantar fasciitis, an outstanding safety profile, and a mechanism—adenosine A2A-mediated suppression of TNF-α and IL-1β with VEGF-driven neovascularization and nucleoside salvage—that maps unusually well onto the hypoxic, acidic, sensitizer-rich microenvironment of the myofascial trigger point.

For myofascial pain syndrome of the trapezius and rhomboid specifically, neither agent can presently be recommended as standard care, and clinicians choosing between them are extrapolating. On the basis of current preclinical and indirect evidence, PDRN’s mechanism aligns more closely with the biochemical features of the MTrP microenvironment; however, whether this congruence translates into superior clinical outcomes is unknown. PRP currently holds a broader and more methodologically robust clinical evidence base, though this advantage is not specific to the myofascial indication. That this tension remains unresolved after a decade of clinical use in both Korea and Europe reflects a literature dominated by small, uncontrolled, single-agent studies. Resolving it requires ultrasound-guided, muscle-specific, placebo-controlled head-to-head trials with characterized injectates and mechanistic endpoints. Until those exist, the honest position is that both agents are promising, and neither is proven.

## Figures and Tables

**Table 1:** Mechanistic and pharmaceutical comparison of platelet-rich plasma (PRP) and polydeoxyribonucleotide (PDRN).

Domain	PRP	PDRN
Source	Autologous whole blood [8]	Salmonid (*O. mykiss*/*O. keta*) sperm DNA, purified [9]
Active principle	Multiple α-granule growth factors (PDGF, TGF-β, VEGF, IGF-1, EGF, FGF) plus leukocyte-derived mediators [8,19]	Deoxyribonucleotide polymers, 50–1500 kDa [9]
Primary molecular target	Multiple receptor tyrosine kinases; no single defined target [8]	Adenosine A2A receptor [9,21,22]
Secondary mechanism	Fibrin scaffold; chemotaxis of progenitor cells [8]	Nucleoside salvage pathway [9,23]
Effect on pro-inflammatory cytokines	Variable; LR-PRP may increase early TNF-α/IL-1β signalling [8,19,20]	Consistent reduction of TNF-α and IL-1β [21,22]
Angiogenic effect	VEGF-mediated, growth-factor dependent [8]	VEGF upregulation via A2A [9,21,23]
Standardization	Poor; no validated classification; kit- and patient-dependent [8,19]	High; defined molecular weight range and manufacturing process [9]
Immunogenicity	None (autologous)	Negligible; protein/peptide-free preparation [9]
Preparation requirement	Venipuncture, centrifugation, ~15–30 min chairside processing [8]	Off-the-shelf ampoule; no processing
Dose reproducibility	Low (inter- and intra-patient variability) [8]	High
Documented safety concerns	Post-injection pain flare; theoretical catabolic effect of leukocytes intra-articularly [20]	Excellent tolerability in large post-marketing surveillance [9]
Mechanistic fit to MTrP milieu (hypoxia, acidosis, TNF-α/IL-1β elevation) [10,11]	Partial: pro-angiogenic, but cytokine effect direction uncertain	High: anti-inflammatory, pro-angiogenic, substrate-supplying

**Table 2:** Principal clinical studies of PRP and PDRN in myofascial pain syndrome and soft-tissue musculoskeletal disorders.

Study (Ref)	Agent	Design	n	Indication/Target	Comparator	Main Finding
Agarwal et al. [29]	PRP	RCT, double-blind	30	Masseter MTrP (MPS)	Dry needling	PRP superior for pain and satisfaction to 6 months
Rani et al. [30]	PRP	RCT	22	Masticatory MPS	Dry needling	Both improved; PRP superior at 4 and 12 weeks
Sakalys et al. [31]	PRP (PRGF)	RCT	50	Masticatory muscle MPS	Lidocaine	PRGF injection tested for pain relief and muscle regeneration
Yilmaz et al. [32]	PRP	RCT	—	Masseter MTrP	Botulinum toxin, local anesthetic	PRP compared against the full range of injectates
Chou et al. [27]	Dextrose (comparator context)	Retrospective series	—	Chronic MPS	—	US-guided injection feasible and effective
Metin Ökmen et al. [5]	Local anesthetic (delivery comparison)	RCT, double-blind	—	Rhomboid vs. trapezius MPS	Trapezius injection	Rhomboid-targeted injection superior
Yoon et al. [33]	PDRN	Case-controlled retrospective	106	Chronic rotator cuff disease	Conservative care	Improved SPADI, VAS, analgesic use to 3–6 months
Ryu et al. [34]	PDRN	Prospective (prolotherapy protocol)	—	Rotator cuff tendinopathy	—	Pain improvement reported
Do et al. [35]	PDRN	Prospective pilot, US-monitored	—	Partial-thickness supraspinatus tear	—	Pain and sonographic improvement
Gwak et al. [28]	PDRN	Meta-analysis (1 RCT + 3 retrospective)	—	Tendon/ligament pain	Mixed	SMD −1.43 for pain; −2.34 in rotator cuff subgroup
Kim and Chung [40]	PDRN	RCT	40	Chronic plantar fasciitis	Normal saline	PDRN superior on VAS and MOXFQ at 4 and 12 weeks
Lee et al. [41]	PDRN	RCT	44	Plantar fasciitis	Corticosteroid	Corticosteroid better at 2 and 6 weeks; equivalent at 6 months

MPS, myofascial pain syndrome; MTrP, myofascial trigger point; MOXFQ, Manchester–Oxford Foot Questionnaire; PRGF, Plasma Rich in Growth Factors; RCT, randomized controlled trial; SMD, standardized mean difference; SPADI, Shoulder Pain and Disability Index; US, ultrasound; VAS, visual analog scale.

**Table 3:** Comparative evidence position of PRP and PDRN across musculoskeletal indications.

Indication	PRP: Strength of Evidence	PDRN: Strength of Evidence	Comparative Position
Trapezius/rhomboid MPS	Very low—no dedicated RCT [5,26,27]	Very low—no dedicated RCT [28,33]	Neither established; direct comparison absent
Masticatory MPS	Low–moderate—3 small RCTs [29,30,31]	Absent	PRP has the only direct MPS evidence
Rotator cuff tendinopathy	Moderate—RCTs and network meta-analysis [36]	Low—retrospective and pilot studies, meta-analysis of weak designs [28,33,34,35]	PRP better studied; PDRN effect estimates larger but less reliable
Lateral epicondylitis	Moderate—RCTs vs. corticosteroid [38,39]	Low—case series and combination protocols	PRP better studied
Plantar fasciitis	Moderate—RCTs vs. corticosteroid [42]	Moderate—placebo-controlled and active-comparator RCTs [40,41]	Comparable; PDRN has a placebo-controlled trial
Knee osteoarthritis	High—multiple Level 1 meta-analyses [45,46,47,48]	Moderate—meta-analysis of 5 RCTs vs. HA [43,44]	PRP stronger; both confounded by saline effect [49]
Acute muscle injury	Moderate but negative—blinded placebo-controlled RCT [50,51]	Absent	PRP not supported

HA, hyaluronic acid; MPS, myofascial pain syndrome; RCT, randomized controlled trial.

**Table 4:** Practical considerations for clinical use of PRP and PDRN in myofascial and musculoskeletal indications.

Consideration	PRP	PDRN
Chairside workflow	Venipuncture, centrifugation, product transfer; adds time and disposable cost [8]	Ready-to-use ampoule; no processing
Typical regimen in MPS/tendon studies	Single or 2–3 injections at 1–4 week intervals [29,30,33]	Weekly injections × 3, or 3–4 sessions at 1–2 week intervals [40,41]
Imaging guidance	Recommended; essential for deep targets such as rhomboid [5,6,18]	Same
Dose reporting in the literature	Rarely characterized (platelet concentration, leukocyte content usually unstated) [8,19]	Usually stated as mg per mL and total volume [33,40,41]
Contraindications	Thrombocytopenia, active infection, anticoagulant/antiplatelet considerations [8]	Fish-protein hypersensitivity (theoretical; product is protein-free) [9]
Adverse event profile	Post-injection pain flare; leukocyte-dependent intra-articular reactions [20]	Excellent tolerability in large-scale surveillance [9]
Advantage over corticosteroid	Avoids tendon and fascial compromise [38,39]	Avoids tendon and fascial compromise; no metabolic effect [39,41]
Principal weakness	Product heterogeneity undermines reproducibility [8,19]	Evidence base dominated by small, non-randomized studies [28,33]

## Data Availability

No new data were created or analysed in this study. Data sharing is not applicable to this article.
